# Anterior deltoid atrophy after reverse shoulder arthroplasty: a preliminary prospective study on surgical approach and neurophysiological correlates

**DOI:** 10.1007/s00264-026-06804-4

**Published:** 2026-04-23

**Authors:** Yaiza Lopiz, Alberto Rodrígiez-González, Eduardo Ossuna-Juntadez, Carlos García-Fernández, Susana Martín-Albarrán, Fernando Marco

**Affiliations:** 1 Shoulder and Elbow Unit, Department of Traumatology and Orthopaedic Surgery, Clínico San Carlos Hospital, Madrid, Spain; 2 Surgery Department, Complutense University, Madrid, Spain; 3 Clinical Neurophysiology Department, Clínico San Carlos Hospital, Madrid, Spain

**Keywords:** Reverse shoulder arthroplasty, Deltoid atrophy, Axillary nerve, Electromyography, Surgical approach, Rotator cuff arthropathy

## Abstract

**Purpose:**

To assess the incidence of anterior deltoid atrophy following reverse total shoulder arthroplasty (RTSA) for rotator cuff arthropathy (RCA), to investigate its association with the surgical approach and neurophysiological injury of the anterior branch of the axillary nerve, and to determine its impact on postoperative shoulder flexion.

**Methods:**

Prospective observational cohort study of 31 patients (mean age 77.9 ± 5.4 years; 85% female) with RCA undergoing RTSA at a single tertiary centre (2014–2017). Two approaches were used: deltopectoral (DP, *n* = 20) and superolateral (SL, *n* = 11). Neurophysiological evaluation (electroneurography + quantitative needle EMG) of the axillary and suprascapular nerves was performed preoperatively and at three and six months postoperatively by a single experienced neurophysiologist. Anterior deltoid atrophy was assessed at 12 months using a pre-specified standardised clinical inspection protocol: visible anterior deltoid contour concavity at rest, confirmed on active elevation against gravity, graded as present or absent by a single blinded examiner. Convergent support was provided by the observed difference in shoulder flexion between groups and by the EMG data. Shoulder flexion and the Constant-Murley Score (CMS) were recorded at baseline and 12 months.

**Results:**

Preoperative axillary nerve injury was present in 77.4% of patients, predominantly affecting the anterior branch (48.4%). Acute postoperative axillary nerve injury occurred in 25.8% of the overall cohort. At 12 months, anterior deltoid atrophy was identified in 13/31 patients (41.9%), with a significantly higher rate in the SL group (72.7% vs 25%; p = 0.021). The rate of acute postoperative injury to the anterior axillary nerve branch did not differ significantly between patients with and without deltoid atrophy (23.1% vs 22.2%; p = n.s.). Patients with atrophy achieved a mean anterior flexion of 115° (SD 8.7°) versus 137° (SD 7.4°) in those without (difference 22°; 95% CI 1.5–31.2; p = 0.066; Cohen's d = 0.87). Both groups improved significantly from baseline.

**Conclusion:**

Anterior deltoid atrophy is common after RTSA (42%) and is significantly associated with the superolateral approach. The absence of a neurophysiological correlate is consistent with a mechanical aetiology related to deltoid reinsertion technique, although causality cannot be established from this observational study. These findings generate a testable hypothesis warranting prospective evaluation of bony acromial flap reinsertion in future comparative studies.

## Introduction

Reverse total shoulder arthroplasty (RTSA), introduced by Grammont in the 1980s, has become the standard surgical treatment for rotator cuff arthropathy (RCA), a condition characterised by massive irreparable rotator cuff tears associated with glenohumeral arthritis and superior humeral head migration [1, 2]. Its fundamental biomechanical principle—medialization and inferior displacement of the centre of rotation—recruits the deltoid as the primary actuator of shoulder elevation, compensating for the dysfunctional rotator cuff [3].

The deltoid muscle is consequently paramount for functional recovery after RTSA. It provides approximately 50% of the force required for arm elevation in the scapular plane under normal conditions; in the rotator cuff-deficient shoulder with an RTSA implant, it becomes the sole source of abduction moment [4, 5]. Any compromise of deltoid integrity—whether neurological, mechanical, or atrophic—may therefore have a disproportionate impact on outcomes. Recent evidence further suggests that RTSA itself induces histological changes in the deltoid muscle fibres independently of neurological injury, with a significant reduction in mean muscle fibre area detectable as early as 12 months postoperatively [6].

The axillary nerve, which provides motor innervation to all three deltoid heads, is the peripheral nerve most at risk during shoulder surgery. In the context of RCA, preoperative neurophysiological alterations of the axillary nerve are already highly prevalent, reported in up to 64–77% of patients even before surgery, reflecting chronic denervation from long-standing mechanical impingement and disuse [7, 8]. A recent prospective electrodiagnostic study confirmed these findings and demonstrated that most acute postoperative injuries were transient, with almost complete neurophysiological recovery at six months and little functional impact [9]. At population level, clinically evident nerve injury after primary RTSA is reported in approximately 1.3% of cases, though neurophysiological studies consistently reveal a far higher rate of subclinical injury [10]. The superimposition of surgical trauma on this pre-existing neurological vulnerability may further compromise deltoid function postoperatively.

Two main surgical approaches are used for RTSA: the deltopectoral (DP) approach, which accesses the joint through the internervous plane between the deltoid and pectoralis major, and the superolateral (SL) or transdeltoid approach, which longitudinally splits the anterior and middle deltoid fibres, preserving the subscapularis but potentially exposing the anterior deltoid and its axillary nerve supply to direct injury [11, 12]. A recent meta-analysis of comparative studies found no significant difference in overall functional outcomes between the two approaches, but identified specific complications—including greater instability with DP and higher glenoid component loosening with SL—that influence approach selection [13].

Anterior deltoid atrophy is an under-reported complication of RTSA with potentially devastating functional consequences. Greiner et al. [14] documented a correlation between its presence and lower Constant-Murley scores. A recent narrative review of perioperative deltoid pathologies in RSA confirmed that deltoid integrity is essential for successful outcomes and highlighted the multifactorial nature of deltoid insults, encompassing preoperative axillary nerve dysfunction, intraoperative mechanical disruption, and postoperative biological changes [15]. However, the precise incidence of anterior deltoid atrophy, its neurophysiological substrate, and its relationship to surgical approach have not been prospectively characterised in a dedicated study.

The aims of this study were: (1) to prospectively determine the incidence of anterior deltoid atrophy at 12 months following RTSA for RCA; (2) to analyse its relationship with the surgical approach; (3) to investigate the correlation between clinical atrophy and neurophysiological axillary nerve injury; and (4) to determine the functional impact of atrophy on shoulder flexion and overall outcomes.

## Materials and methods

### Study design and ethics

A prospective, single-centre observational cohort study was conducted at the Shoulder and Elbow Surgery Unit of a tertiary referral academic hospital. The protocol was approved by the institutional Clinical Research Ethics Committee and all procedures were conducted in accordance with the Declaration of Helsinki. Written informed consent was obtained from all participants.

### Participants

Patients with RCA scheduled for RTSA between December 2014 and December 2017 were prospectively enrolled. Inclusion criteria were: (1) pain and functional impairment refractory to conservative treatment; (2) radiographic RCA Hamada grade II–V; (3) suitability for RTSA; and (4) capacity to provide written informed consent. Exclusion criteria included: contralateral shoulder disease; comorbidities predisposing to peripheral neuropathy (diabetes mellitus, alcoholism, demyelinating disorders); prior ipsilateral or contralateral shoulder surgery; and inadequate adherence support. Forty patients were enrolled; nine were lost to follow-up (six withdrew before surgery, two declined postoperative neurophysiology, one died of unrelated causes). The final cohort comprised 31 patients with a minimum follow-up of 24 months (mean 28.4 ± 4.4 months, range 24–36).

### Surgical technique

All procedures were performed in the beach-chair position under interscalene block combined with general anaesthesia. Two prosthetic systems were used: the Delta III (DePuy, Warsaw, IN, USA) and the Lima SMR (Lima Corporate, San Daniele del Friuli, Italy). Approach selection was at the surgeon's discretion:

Deltopectoral (DP) approach: incision from 0.5 cm lateral to the coracoid tip, extending 10–15 cm distally; dissection through the deltopectoral interval with lateral retraction of the cephalic vein; subscapularis tenotomy with tendon tagging for reinsertion.

Superolateral (SL)/transdeltoid approach: longitudinal incision along the anterior acromial border, not exceeding 4 cm distally to remain within the safe zone of the axillary nerve (5–7 cm from the lateral acromial edge) [11]; longitudinal splitting of the deltoid between its anterior and middle thirds; subscapularis preservation. Anterior deltoid reinsertion was performed using transosseous suture at the acromial origin.

### Neurophysiological assessment

Neurophysiological evaluation was performed preoperatively and at three and six months postoperatively by a single experienced neurophysiologist. The study comprised:

1. Motor nerve conduction study (electroneurography): stimulation at Erb's point with surface electrode recording over deltoid and infraspinatus muscles. Abnormality was defined as a > 50% reduction in compound muscle action potential (CMAP) amplitude compared with the contralateral limb.
2. Quantitative needle EMG: analysis of the anterior, middle, and posterior deltoid heads (axillary nerve branches) and the supraspinatus and infraspinatus (suprascapular nerve). Motor unit potential (MUP) morphology and recruitment pattern were assessed at rest and at maximum voluntary effort. Three neurophysiological patterns were classified: (a) acute axonal injury (fibrillations, positive sharp waves, reduced recruitment); (b) chronic axonal injury (polyphasic reinnervation potentials, reduced recruitment); and (c) disuse atrophy (normal MUPs, reduced recruitment, normal spontaneous activity).

### Outcome measures

The primary outcome was the presence or absence of anterior deltoid atrophy at 12 months, defined as visible concavity of the anterior deltoid contour in the relaxed position, confirmed on active arm elevation against gravity. Assessment was performed by a single examiner (the operating surgeon) blinded to all neurophysiological results. Secondary outcomes were: (1) active anterior shoulder flexion (manual goniometer) at baseline and 12 months; (2) active abduction; (3) VAS pain score (0–10); and (4) absolute CMS (aCMS) and relative age- and sex-adjusted CMS (rCMS).

### Statistical analysis

Continuous variables are expressed as mean ± SD with 95% CI where appropriate. Categorical variables as frequencies (%). Differences in atrophy rates between groups were assessed with Fisher's exact test. Intragroup pre-to-post comparisons used the Wilcoxon signed-rank test. Intergroup comparisons used the Mann–Whitney U test. Logistic regression was performed to explore potential predictors of atrophy (surgical approach, age, Hamada grade); given the small sample size and low number of outcome events (13 events across three predictor variables), this model violates the conventional threshold of ≥ 10 events per variable and must be considered descriptive and hypothesis-generating only. Effect sizes for between-group comparisons are reported as Cohen's d. A *p* value < 0.05 was considered statistically significant. Analyses were performed with SPSS Statistics v.25.0 (IBM Corp., Armonk, NY, USA).

## Results

### Patient characteristics

The 31-patient cohort had a mean age of 77.9 ± 5.4 years (range 67–86); 29 (85%) were female. The right shoulder was affected in 77.4% and the dominant limb in 84%. The most frequent comorbidity was hypertension (74.2%). ASA class was II in 67.7%. All patients had preoperative MRI fatty infiltration classified as Fuchs grade III. Hamada distribution: grade II 67.8%, grade III 12.9%, grade IV 12.9%, grade V 6.4%. Twenty patients (64.5%) underwent DP approach and 11 (35.5%) SL approach.

### Preoperative neurophysiological findings

Preoperative neurophysiological abnormalities were detected in 26/31 patients (83.9%). Axillary nerve injury was present in 77.4% of cases (24/31), comprising: anterior branch—51.6% normal, 35.5% chronic injury, 12.9% disuse; middle branch—71% normal, 19.4% chronic, 9.6% disuse; posterior branch—25.8% normal, 9.7% chronic, 64.5% disuse (Table 1). Suprascapular nerve injury was present in 45.2% (14/31).

**Table 1:** Preoperative neurophysiological findings by axillary nerve branch (*n* = 31)

Axillary nerve branch	Normal n (%)	Chronic injury n (%)	Disuse n (%)
Anterior	16 (51.6%)	11 (35.5%)	4 (12.9%)
Middle	22 (71.0%)	6 (19.4%)	3 (9.6%)
Posterior	8 (25.8%)	3 (9.7%)	20 (64.5%)

*Chronic injury* polyphasic reinnervation potentials, *Disuse* reduced recruitment with normal MUP morphology.

### Postoperative axillary nerve injury

Acute postoperative neurophysiological injury was identified in 11/31 patients (35.5%) at the three month EMG. By nerve, eight patients (25.8%) had acute axillary nerve injury and six (19.4%) had acute suprascapular nerve injury; three patients had combined injury to both nerves. Within the axillary nerve branches, acute injury predominantly affected the anterior branch (22.6%, 7/31), with a much lower rate in the middle branch (3.2%, 1/31) and no cases in the posterior branch (Table 2). At the six month follow-up EMG, the majority of acute injuries showed improvement: 5/7 anterior branch injuries (71.4%) improved, with two (28.6%) showing no electrophysiological change.

**Table 2:** Acute postoperative axillary nerve branch injury at 3-month EMG (*n* = 31)

Branch	No acute injury n (%)	Acute postoperative injury n (%)	Recovery at 6 months
Anterior	24 (77.4%)	7 (22.6%)	5/7 improved (71.4%)
Middle	30 (96.8%)	1 (3.2%)	1/1 improved (100%)
Posterior	31 (100%)	0 (0%)	—

Acute injury defined as new postoperative fibrillations, positive sharp waves, or reduced recruitment compared with preoperative study.

### Incidence of anterior deltoid atrophy and relationship with surgical approach

**Fig. 1:**
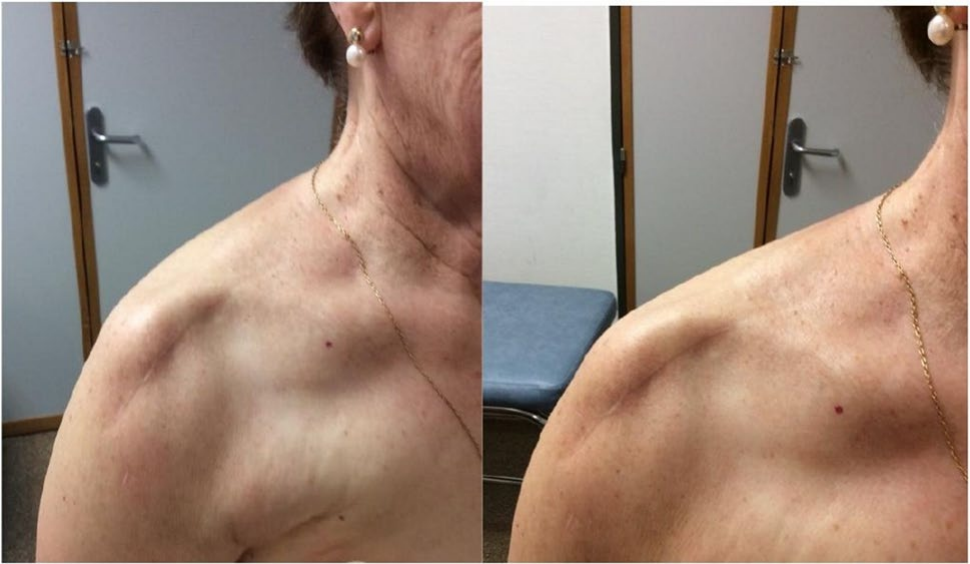
Clinical photographs of a patient with anterior deltoid atrophy at 12 months following reverse total shoulder arthroplasty. The characteristic anterior contour concavity is visible in both anterolateral (left) and lateral (right) views. Reproduced with written informed patient consent

**Fig. 2:**
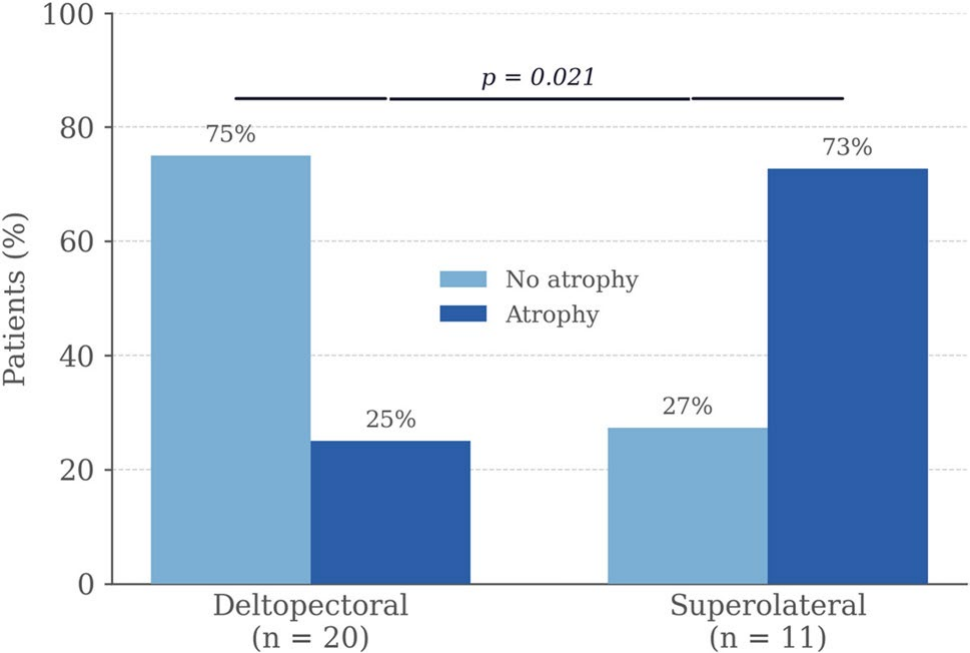
Anterior deltoid atrophy at 12 months by surgical approach (Fisher's exact test)

Representative clinical photographs of anterior deltoid atrophy at 12 months postoperatively are shown in Fig. 1. At the 12-month clinical review, anterior deltoid atrophy was detected in 13/31 patients (41.9%). The rate was significantly higher in the SL group (8/11, 72.7%) than in the DP group (5/20, 25%; p = 0.021, Fisher's exact test). These findings are illustrated in Fig. 2.

### Neurophysiological correlates of deltoid atrophy: dissociation between nerve injury and atrophy

**Fig. 3:**
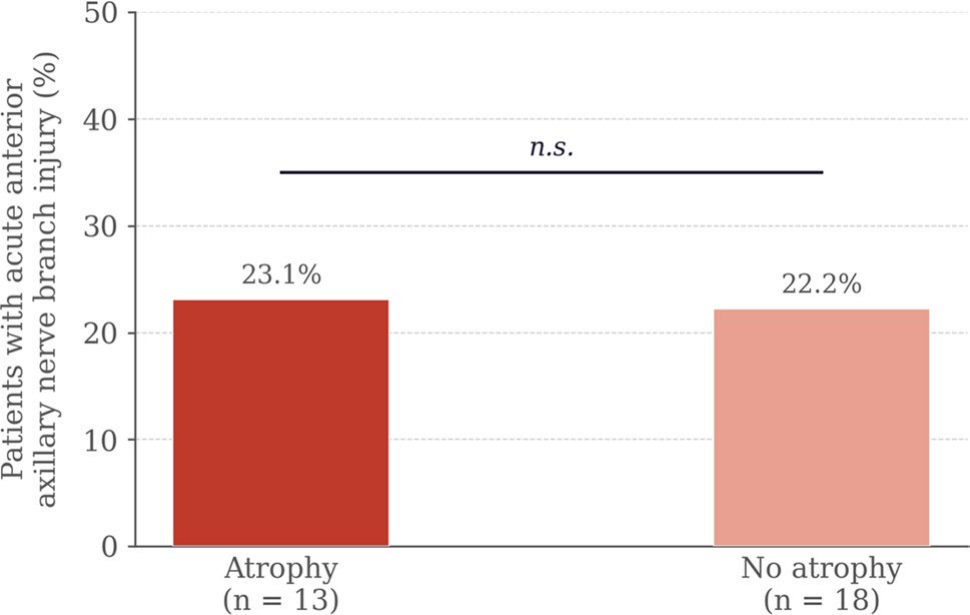
Rate of acute postoperative anterior axillary nerve branch injury according to deltoid atrophy status (n.s.: not significant, Fisher's exact test; acute injury defined at 3-month EMG)

The rate of acute anterior branch injury did not differ significantly between patients who developed atrophy (23.1%) and those who did not (22.2%; *p* = n.s., Fisher's exact test), as illustrated in Fig. 3.

### Impact on shoulder flexion

**Fig. 4:**
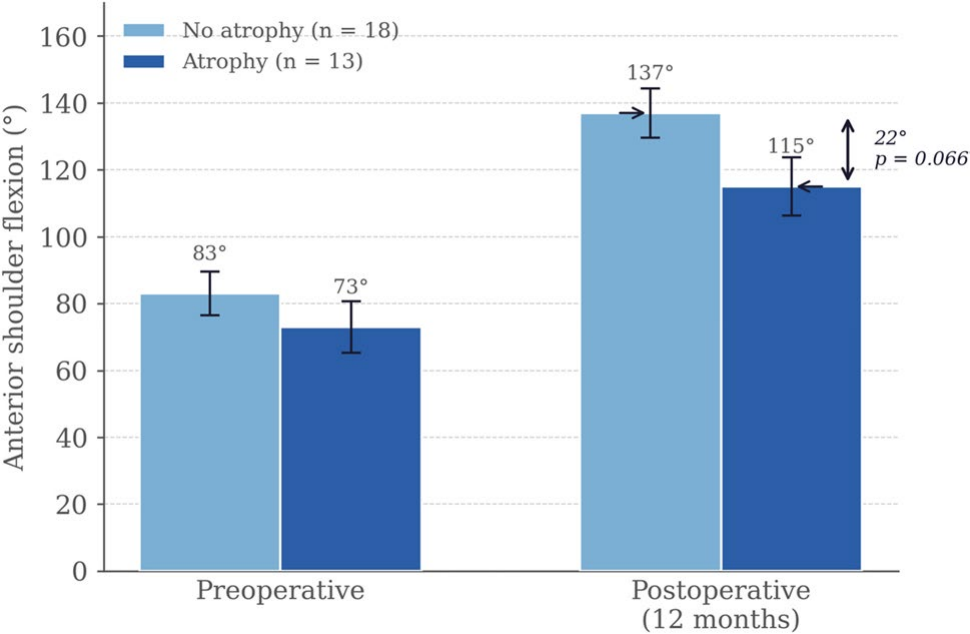
Anterior shoulder flexion (mean ± SD) by deltoid atrophy status, preoperatively and at 12 months. p intra: within-group comparison (Wilcoxon); p inter: between-group comparison (Mann–Whitney U)

Patients with anterior deltoid atrophy achieved a mean postoperative flexion of 115° ± 8.7° compared with 137° ± 7.4° in those without atrophy (difference 22°; 95% CI 1.5–31.2; *p* = 0.066; Cohen's d = 0.87, a large effect size by conventional thresholds). Both groups showed significant intragroup improvement from baseline (atrophy: 73° → 115°, *p* = 0.001; no atrophy: 83° → 137°, *p* < 0.001). No significant intergroup difference was found in the pattern of flexion evolution (*p* = 0.440). Post-hoc power analysis indicated that the study had approximately 43% power to detect the observed difference at α = 0.05; this secondary outcome was therefore underpowered and should be interpreted as an exploratory signal only. Results are shown in Fig. 4.

### Overall functional outcomes by surgical approach

All patients improved significantly from preoperative values regardless of approach. The SL group showed marginally lower postoperative scores for flexion (120° vs 132°), abduction (115° vs 118°), rCMS (81.3 vs 90.3), and VAS (2.1 vs 2.0), but none of these differences reached statistical significance (*p* = 0.441, 0.363, 0.185, and 0.499, respectively).

## Discussion

This prospective exploratory study reports three principal findings: (1) anterior deltoid atrophy occurs in approximately 42% of patients at 12 months following RTSA for RCA; (2) it is significantly more frequent with the superolateral surgical approach; and (3) the rate of acute postoperative axillary nerve injury does not differ between patients who develop atrophy and those who do not, a finding consistent with—though not conclusive of—a predominantly mechanical aetiology.

The high incidence of preoperative neurophysiological abnormalities (83.9%) confirms findings from our group's prior published work [8, 9] and from the wider literature. The posterior branch of the axillary nerve was most severely affected preoperatively (74.2% showing disuse changes), likely reflecting chronic external compression by the migrated humeral head. The anterior branch showed the highest rate of acute postoperative injury (22.6%), consistent with its anatomical vulnerability during anterior shoulder dissection. That acute anterior branch injury resolved in 71% of cases at six months aligns with the predominantly neurapraxic nature of perioperative nerve injury in elective arthroplasty, as reported in large series and confirmed by systematic review [10, 16] and confirmed by systematic review [17, 18]. The anatomical basis of this vulnerability has been characterised in cadaveric studies demonstrating that humeral descent during implantation alters the course and tension of the axillary nerve, potentially contributing to transient neurapraxic injury [19].

The central and most novel finding is the absence of a significant relationship between neurophysiological axillary nerve injury and clinical deltoid atrophy. Despite a 22.6% rate of acute anterior branch injury—which might intuitively be expected to predispose to atrophy—the prevalence of atrophy was not higher in patients with confirmed neurophysiological injury. One plausible, though unconfirmed, interpretation of this neurophysiological dissociation is that atrophy in the SL group may reflect a problem with the mechanical reattachment of the anterior deltoid to its acromial origin rather than axillary nerve injury; however, this hypothesis cannot be tested from our data and should be considered speculative. These observations are consistent with, but do not establish, a predominantly mechanical aetiology. The significant association between the SL approach and atrophy (72.7% vs 25%; *p* = 0.021), in contrast to the absence of significant differences in axillary nerve injury rates between approaches (SL 36.4% vs DP 35%), is consistent with this interpretation but does not exclude confounding by unmeasured factors. It is also consistent with the histological evidence of deltoid muscle fibre changes following RTSA that occur independently of neurological injury [7]. Crucially, this is a non-randomised observational study, and important confounders—including individual anatomical variation, tissue quality related to age, case complexity, and the specific deltoid reinsertion technique employed—were not systematically controlled. These factors may independently contribute to atrophy and cannot be excluded as alternative explanations. The association between approach and atrophy should therefore not be interpreted as causal.

Several lines of published evidence are consistent with this interpretation, although none provide direct comparative data. Authors employing the SL approach with a bony acromial flap—preserving the deltoid's bony insertion on an acromial fragment rather than relying on soft-tissue transosseous suture repair—have not described this complication, raising the hypothesis that the reinsertion technique is a modifiable risk factor [13]. Whatley et al. [20] reported postoperative deltoid rupture in patients with prior mini-open rotator cuff repair, suggesting compromised deltoid attachment as a contributing mechanism. Linberg et al. [21] described intraoperative anterior deltoid avulsion requiring clavicular fixation. Collectively, these reports support the hypothesis that bony-interface failure, rather than neural injury, may drive anterior deltoid atrophy in this setting—but direct comparative evidence from randomised studies is lacking.

The deltoid's functional importance in this population cannot be overstated. With the rotator cuff non-functional, the deltoid is the sole provider of abduction moment in RTSA [4], and severe deltoid dysfunction in revision shoulder surgery has been reported in up to 92% of cases when proximal deltoid detachment is performed [16]. The 22° difference in anterior flexion between the atrophy and non-atrophy groups (115° vs 137°; *p* = 0.066; Cohen's d = 0.87) did not reach statistical significance. Post-hoc power analysis indicates that the study had approximately 43% power to detect this difference at α = 0.05, confirming that this secondary outcome was underpowered (estimated requirement: ~ 50 patients per group). This result should therefore be interpreted as a clinically plausible exploratory signal requiring confirmation in a larger study, not as a demonstrated functional effect. The large effect size (Cohen's d = 0.87) nevertheless suggests that a clinically meaningful difference may exist and warrants prospective evaluation in an adequately powered study. These observations are consistent with the findings of Greiner et al. [14], who demonstrated lower Constant scores in patients with postoperative deltoid atrophy.

Our overall functional data—showing similar improvement in both DP and SL approach groups without statistically significant differences—are consistent with the findings of Lädermann et al. [12], who found only a 10° non-significant difference in flexion between approaches in 58 patients, with the French SOFCOT multicentre study (*n* = 527) [22], and with the meta-analysis by Seok et al. [13], which found no significant difference in functional outcomes between the anterosuperior and deltopectoral approaches across six comparative studies. Taken together, the approaches appear functionally equivalent at population level, but the SL approach carries a significantly higher risk of anterior deltoid atrophy that may be preventable with modification of the reinsertion technique.

This study has several limitations that must be acknowledged explicitly. First, the sample size (*n* = 31) is insufficient for definitive conclusions on atrophy-related secondary outcomes. All functional comparisons must therefore be regarded as exploratory signals only. The logistic regression analysis is additionally underpowered, with 13 events across three predictor variables, violating the minimum threshold of ≥  ten events per variable; its findings are strictly descriptive. Second, the surgical approach was not randomised, introducing potential selection bias; unmeasured factors including surgeon preference, case complexity, and patient anatomy may have influenced both approach selection and outcomes, and the association between the SL approach and atrophy cannot be interpreted as causal. Third, important confounders—including individual variation in deltoid reinsertion technique, tissue quality, and surgeon experience—were not systematically recorded or controlled, limiting causal inference. Fourth, the primary outcome (anterior deltoid atrophy) was assessed by clinical inspection rather than objective imaging. Although a pre-specified binary criterion was applied by a single blinded examiner, this approach has specific limitations: inter-observer reliability was not formally assessed; binary grading does not capture the full spectrum of atrophy severity; subclinical degrees of atrophy may have been missed; and ultrasound-measured deltoid thickness or MRI-based volumetry would provide quantitatively superior and more reproducible measures for future studies. The convergent support from functional and neurophysiological data provides indirect but not definitive evidence that the clinical assessment captured a meaningful biological difference. Fifth, EMG was not performed beyond 6 months postoperatively, leaving open the possibility of later-onset neurophysiological changes contributing to atrophy at 12 months; subclinical denervation beyond our observation window cannot be excluded. Sixth, the exclusively elderly female cohort (mean 77.9 years, 85% female) substantially limits generalisability to younger patients, males, and populations with different RCA severity distributions. The single-centre design and use of two prosthetic systems further limit external validity.

Despite these limitations, this prospective study generates a specific and testable hypothesis: that anterior deltoid atrophy following the superolateral approach to RTSA is driven primarily by mechanical disruption at the acromial deltoid origin rather than by axillary nerve injury. If confirmed in larger, controlled studies, this would have direct surgical implications, as the bony acromial flap reinsertion technique—already described by several groups [20, 21]—would represent a straightforward modification to reduce this complication. Future prospective comparative studies with objective atrophy measurement, randomised approach allocation, and systematic recording of reinsertion technique are needed to test this hypothesis.

## Conclusion

Anterior deltoid atrophy is a frequent complication following RTSA for RCA, identified in 42% of patients at 12 months in this prospective series. It is significantly associated with the superolateral surgical approach and is accompanied by a clinically plausible, though statistically non-significant, 22° reduction in shoulder flexion. The absence of a neurophysiological correlate between axillary nerve injury and deltoid atrophy is consistent with a mechanical aetiology related to deltoid reinsertion at the acromial origin, although causality cannot be established from this observational study.

## Data Availability

The datasets generated and analysed during the current study are not publicly available due to patient confidentiality constraints and the terms of the approved ethics protocol. De-identified data are available from the corresponding author upon reasonable request and subject to institutional data sharing agreements.
